# Dual-responsive doxorubicin-loaded nanomicelles for enhanced cancer therapy

**DOI:** 10.1186/s12951-020-00691-6

**Published:** 2020-09-24

**Authors:** Xinyi Zhang, Tiantian Zhu, Yaxin Miao, Lu Zhou, Weifang Zhang

**Affiliations:** 1 Department of Pharmacy/Respiratory Diseases, The Second Affiliated Hospital of Nanchang University, Nanchang, 330006 China; 2 Teaching and Research Office of Clinical Pharmacology, College of Pharmacy, Xinxiang Medical University, Xinxiang, 453003 China; 3 Medical College of Nanchang University, Nanchang, 330031 China

**Keywords:** Doxorubicin, Reactive oxygen species, Charge-reversal, Lung cancer

## Abstract

**Background:**

The enhancement of tumor retention and cellular uptake of drugs are important factors in maximizing anticancer therapy and minimizing side effects of encapsulated drugs. Herein, a delivery nanoplatform, armed with a pH-triggered charge-reversal capability and self-amplifiable reactive oxygen species (ROS)-induced drug release, is constructed by encapsulating doxorubicin (DOX) in pH/ROS-responsive polymeric micelle.

**Results:**

The surface charge of this system was converted from negative to positive from pH 7.4 to pH 6.8, which facilitated the cellular uptake. In addition, methionine-based system was dissociated in a ROS-rich and acidic intracellular environment, resulting in the release of DOX and α-tocopheryl succinate (TOS). Then, the exposed TOS segments further induced the generation of ROS, leading to self-amplifiable disassembly of the micelles and drug release.

**Conclusions:**

We confirms efficient DOX delivery into cancer cells, upregulation of tumoral ROS level and induction of the apoptotic capability in vitro. The system exhibits outstanding tumor inhibition capability in vivo, indicating that dual stimuli nano-system has great potential to function as an anticancer drug delivery platform.
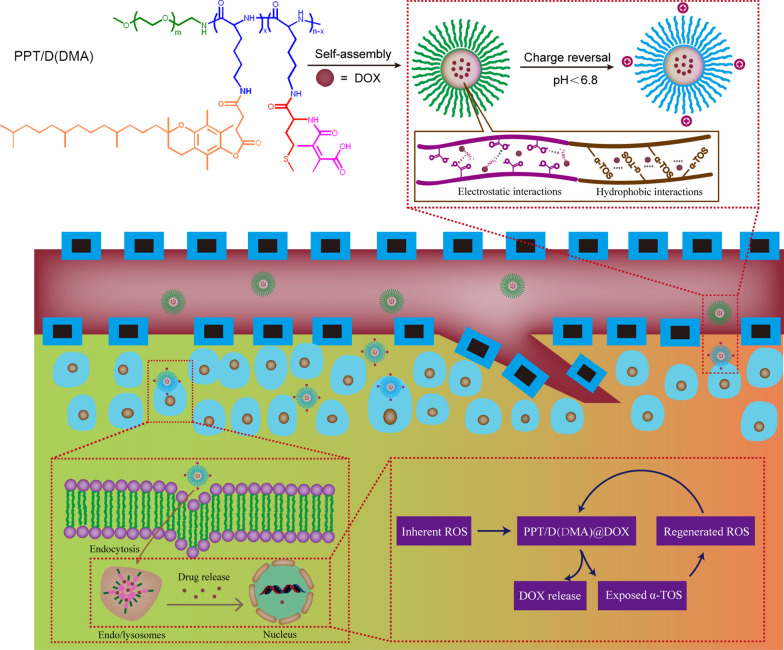

## Background

Plenty of smart drug delivery systems have been proposed to improve the therapeutic efficacy and reduce undesirable side effects through achieving “on demand” drug release at tumor site under unique internal or external stimuli including pH gradient [1–6], intracellular reductive agents [7, 8], peculiar enzymes [9, 10], ROS [11, 12], and so on. However, the stimuli levels of the tumor extracellular microenvironments are particularly subtle, such as relatively low ROS concentration even under pathological conditions, which accordingly imposes strict demand for the sensitivity of the materials responsive to the tumor microenvironments [13–15]. Besides, the poor uptake of tumor cells and incomplete drug release are extra two critical challenges hindering the clinical translation of polymeric drug [6, 15]. Tremendous efforts had been devoted to the development of stimuli-responsive tumor-targeted drug delivery systems to deal with aforementioned dilemma. On one hand, pH-dependent charge conversion strategy is utilized to construct polymeric drug, which maintains their stealth features during circulation and then undergoes a charge reversal process for achieving enhanced tumor cells’ internalization once exposed to tumor relative acidic environment [15–18]. On the other hand, polymeric drug with ROS generation capability will be an effective approach to copying with insufficient ROS concentration for activating the complete drug release [13–15, 19].

Inspired by above implement, we designed a self-amplifiable drug release system with charge reversal ability by loading DOX in a polymeric micelle methoxyl poly(ethylene glycol)-*block*-poly(l-lysine)-*graft*-α-tocopheryl succinate and methionine modified with dimethylmaleic anhydride (denoted as PPT/D(DMA)@DOX).

**Scheme 1:**
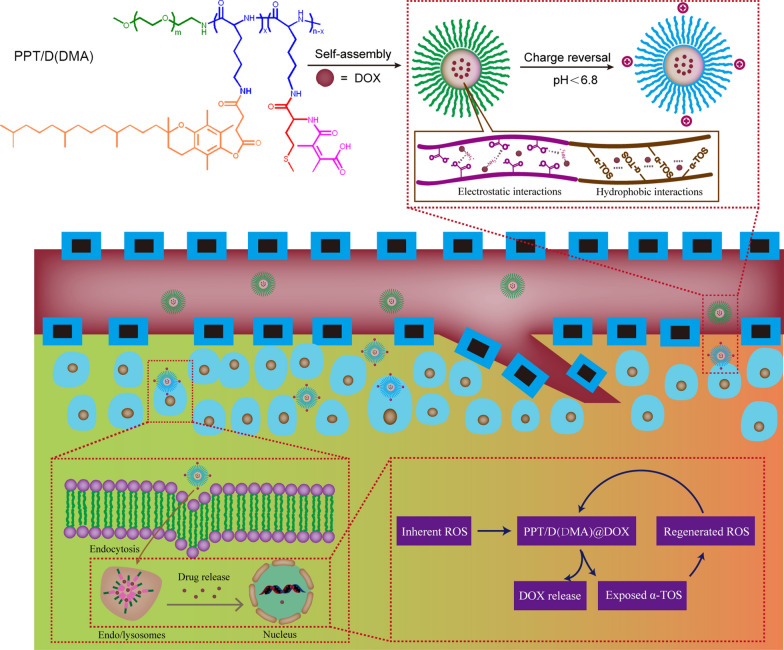
Schematic illustration of the successive behaviors of the multifunctional micelle of the charge reversal PPT/D(DMA)@DOX system with self-amplifiable drug release for tumor therapy in vivo

Firstly, a moderate amount of DMA was conjugated to the backbone of the polymer, endowing the pH-triggered charge reversal property. The pH-dependent charge reversal delivery systems remained negatively charged under physiological environment (pH 7.2–7.4), which facilitates reduced nonspecific interactions with serum components. Under tumor microenvironment (pH 6.2–6.9), they are converted to positive charge, achieving charge reversal to enhance targeted tumor uptake [6, 15]. Secondly, TOS, an analogue of vitamin E, rapidly generates ROS in cells after interacting with mitochondrial respiratory complex II and interfering the electron transportation chain in mitochondria [13]. Thioether groups in poly-methionine segments are changed to hydrophilic sulfoxide groups in cancer cells due to an inundation of ROS [11, 12, 20]. We hypothesized that ROS concentration under pathological conditions can induce the less decomposition of the polymeric drug by the phase transitions and then exposed TOS segment interacted with mitochondria in tumor cells to generate additional ROS. In other words, the intracellular ROS would be self-regenerated and amplified. Finally, TOS is less toxic towards normal cells and shows specific anticancer activities to various cancers [21]. DOX was encapsulated by hydrophobic interaction with TOS and methionine and electrostatic attraction with DMA. Thus, we hypothesized that PPT/D(DMA)@DOX system could effectively enhanced tumor therapeutic efficacy with self-amplifiable drug release. In this study, ^1^H-NMR was used to confirm the successful synthesis of polymers. Several in vitro and in vivo characteristics were performed. Meanwhile, the indexes indicated a great pH/ROS-sensitive antitumor efficacy of micelle, which would be potential nanocarrier for lung cancer therapy (Scheme 1).

## Materials and methods

### Materials

Methoxy poly(ethylene glycol) amine (mPEG-NH_2_, *M*_n_ = 5000), Polyethylene-polypropylene glycol (PLGA-mPEG, *M*_n_ = 8000), doxorubicin hydrochloride (DOX) and α-tocopheryl succinate (TOS) were purchased from Macklin Company, China. *N*(ε)-benzyloxycarbonyl-l-lysine (Cbz-lysine) and l-methionine was purchased from Energy Chemical, China. Dimethylmaleic anhydride (DMA) was purchased from Bidepharm, China. l-lysine-*N*-carboxyanhydride (Cbz-Lys-NCA) were synthesized by the Fuchs-Farthing method using bis(trichloromethyl) carbonate (triphosgene) as reported earlier [6, 22]. Propidium iodide and 4′, 6-diamidino-2-phenylindole dihydrochloride (DAPI) were purchased from Sigma Company. Annexin V-FITC Apoptosis Detection Kit was purchased from Kengen. All other reagents and solvents were purchased from Sinopharm Chemical Reagent Co., Ltd., China.

### Synthesis of methoxyl poly(ethylene glycol)-*block*-poly(l-lysine) (mPEG-*b*-PLL)

Firstly, poly(ethylene glycol)-*block*-poly(*N*(ε)-benzyloxycarbonyl-l-lysine) (mPEG-*b*-PLL(Z)) was synthesized by ring open polymerization (ROP) of Lys(Z)-NCA in DMF using mPEG-NH_2_ as initiator and the deprotection of benzyl groups as described earlier [22, 23]. Typically, dried mPEG-NH_2_ (1.0 g, 0.2 mmol) and Cbz-Lys-NCA (1.8 g, 6.0 mmol) were added in a 50 mL dried glass reactor with 30 mL of DMF. Stirred at 25 °C for 3 days, the reaction mixture was poured into 150.0 mL of ice ether for three times to precipitate pure mPEG-*b*-PLL(Z) polymers (Yield: 88.6%). The degree of polymerization (DP) of mPEG-*b*-PLL(Z) was determined to be 30 by ^1^H NMR spectra (Additional file 1: Figure S1). The number-average molecular weights (*M*_n_) of mPEG-*b*-PBLG polymer calculated by ^1^H-NMR was 12870 (mPEG_5000_-*b*-PLL(Z)_7870_). Subsequently, 1.6 g of mPEG-*b*-PLL(Z) was added into 10.0 mL of trifluoroacetic acid (TFA) and HBr/HOAc (0.8 mL) to remove Cbz groups. Stirred for 1 h under ice bath, the mixture was precipitated into 150.0 mL of ice diethyl ether, following being dialyzed (molecular weight cut-off (MWCO) = 3500 Da) against distilled water, and then freeze-dried to obtain mPEG-*b*-PLL product and the yield was 75.6%. The purified product was dried under vacuum at room temperature.

### Synthesis of methoxyl poly(ethylene glycol)-*block*-poly(l-lysine)-*graft*-α-tocopheryl succinate and methionine modified with dimethylmaleic anhydride (PPT/M(DMA))

The *N*-(tert-Butoxycarbonyl)-l-methionine (D(Boc)) was firstly prepared as described previously [24]. Dried mPEG-*b*-PLL (1.0 g, 0.11 mmol), D(Boc) (0.74 g, 3.30 mmol), NHS (1.71 g, 14.85 mmol) and DCC (3.06 g, 14.85 mmol) was dissolved in 30.0 mL of DMSO under nitrogen gas. Stirred at 25 °C for 24 h, the reaction solution was firstly filtered and dialyzed against DMSO and then dialyzed against distilled water, following freeze-dried to obtain methoxyl poly(ethylene glycol)-*block*-poly(l-lysine)-*graft*-methionine (Boc) (PPT/D(Boc)) product (PPD(Boc)). The number of grafted D(Boc) was 24 from ^1^HNMR (Additional file 1: Figure S2). PPD(Boc) (1.15 g, 0.08 mmol), TOS (0.35 g, 0.66 mmol), *N*-hydroxysuccinimide (NHS, 0.15 g, 1.32 mmol) and *N*, *N*′-dicyclohexylcarbodiimide (DCC, 0.27 g, 1.32 mmol) was dissolved in 30.0 mL of DMSO under nitrogen gas. Stirred at 25 °C for 48 h, the reaction solution was firstly filtered dialyzed against DMSO and then dialyzed against distilled water, following being freeze-dried to obtain methoxyl poly(ethylene glycol)-*block*-poly(l-lysine)-*graft*-α-tocopheryl succinate and methionine (Boc) (PPT/D(Boc)) product (Additional file 1: Figure S3). Subsequently, 0.8 g of PPT/D(Boc) was dissolved in 5.0 mL of DCM and 5.0 mL of TFA was added dropwise under ice bath. Stirred for 1 h, the mixture was filtrated and precipitated into 100.0 mL of ice diethyl ether, following dialyzed against distilled water, and then freeze-dried to obtain poly(ethylene glycol)-*block*-poly(l-lysine)-*graft*-α-tocopheryl succinate and methionine (PPT/D) product. The shell was prepared by the reaction between PPT/D and DMA [25, 26]. Briefly, PPT/D and double DMA were dissolved in DMSO, then triethylamine (TEA) and pyridine were added and stirring under nitrogen protection at the room temperature overnight. The mixture was purified by dialysis (MWCO 3500 Da) against DMSO for 24 h, following being dialyzed in dialysis bag (MWCO 10,000 Da) for 24 h to remove DMSO, and lyophilized. For comparison, succinic anhydride (SA)-modified shell (Shell-SA) was prepared in the similar route and denoted as PPT/D(SA). SA modified micelle would not undergo pH-sensitive hydrolysis and thus not offering surface charge reversal as DMA modified one does. PPD was obtained with deprotection of PPD(Boc), modified with DMA without TOS and denoted as PPD(DMA) (Additional file 1: Figure S3). As comparison, PPT/D(SA) was synthesized and characterized as shown in Additional file 1: Figure S4. To investigate the ROS-responsiveness, methionine was incubated with H_2_O_2_ for 0, 4 and 12 h in D_2_O. The chemical changes were recorded by ^1^H NMR in Additional file 1: Figure S5. Methionine and its oxidized product were observed 4 h later. After 12-h incubation, methionine was completely oxidized.

### Micelle preparation and characterization

The solvent exchange method was used to prepare micelles in this study. DOX·HCl (5.0 mg) was dissolved in DMF (1 mL) with TEA (2.6 mg) to remove the HCl of the DOX·HCl and was stirred for 2 h. An amount of PPT/D(DMA) was dissolved in DMF (1 mL), mixed with the above solution and then stirred in dark for another 2 h. Then the solution was added to 5 mL PBS (pH 9.0) by using infusion pump at a constant rate of 2 mL/h and was stirred for another 3 h. After that the solution was loaded into a MWCO 3500 dialysis bag and dialyzed against pH 8.0–9.0 water for 24 h. The obtained solution was mildly centrifuged and the supernatant was stored in 0 °C which would be used directly. The similar process was for other micelles. The DOX loading content was determined by lyophilizing 1.0 mL of the above solution and dissolved the obtained powder in DMSO. The concentration of the DOX-loaded micelles was measured at excitation and emission wavelengths of 485 nm and 550 nm. The loading content (LC) and encapsulation efficiency (LE) of DOX micelles were calculated by the following equations.

1 $$LC \% = \frac{Amount\;of\;drugs\;entrapped\;in\;nanoparticles}{Total\;amount\;of\; nanoparticles} \times 100 \%$$

2 $$EE \% = \frac{Amount\;of\;drugs\;\;entrapped\;in\;nanoparticles}{Initial\;\;amount\;of\;drug\;added} \times 100 \%$$

For the characterizations of the empty micelle and DOX-loaded micelles, the particle sizes, size distributions and zeta-potentials were measured by using a Zetasizer (Malvern 3000HSA). The morphologies of the micelles were identified by transmission electron microscopy (TEM) images obtained by a JEM-2000EXII transmission electron microscope with an accelerating voltage of 200 kV. The morphology changes of micelles were also evaluated when micelles were incubated in different pH solutions or H_2_O_2_ solutions.

### pH-sensitive property of PPT/D(DMA)@DOX

In order to evaluate pH-sensitive property of PPT/D(DMA), the micelle was tested compared with PPT/D(SA). Micelles (500 μg/mL) were incubated in PBS at pH 7.4 and 6.8 for 200 min, respectively. At predetermined intervals, the mean diameter and zeta potentials of the micelles were measured by DLS. To evaluate the pH-sensitive property of PPT/D(DMA)@DOX and PPT/D(SA)@DOX, DOX-loaded micelles were incubated at pH 7.4, 6.8 and 5.5 for 200 min. Average size and zeta potential were recorded by DLS.

### Drug release behavior

To study the ROS responsibility of PPT/D(DMA)@DOX, 3.0 mg of micelles suspended respectively in 1.0 mL of PBS (pH = 7.4) containing various concentrations of H_2_O_2_ (0, 0.1, and 10.0 mM) were sealed in a dialysis bag (MWCO 3.5 kDa). The dialysis tubes were subsequently immersed into glass tube containing 30.0 mL of PBS (pH = 7.4) with same concentrations of H_2_O_2_. The released DOX and TOS were measured by a Lambda Bio40 UV/Vis spectrometer and HPLC, respectively. Likewise, the same concentration of PPT/D(DMA)@DOX and PPT/D(SA)@DOX was immersed in buffer solution with various pH values (pH 7.4, 6.8 and 5.5) at 37 °C under shaking at 100 rpm. The release media were taken out at predetermined times and an equal volume of fresh PBS was added. After that, the amounts of released DOX and TOS were detected.

### Cell culture

Human lung adenoma cell lines A549 were purchased from the American Type Culture Collection (ATCC, Rockville). The cells were cultured in complete Dulbecco’s modified Eagle’s medium (DMEM) supplemented with 10% fetal bovine serum (FBS), 100 U/mL penicillin and 100 U/mL streptomycin and grown in a 37 °C humidified environment containing 5% CO_2_.

### Evaluation of the ROS-responsiveness in cells

ROS level changes in cells were determined with 2′,7-dichlorofluoresceindiacetate (DCFA-DA) dye. A549 cells were seeded onto plates at a density of 1.0 × 10^5^ cells per well. After an incubation for 24 h, the cells were treated with empty micelles (PPT/D(DMA) or PPD(DMA)) and PPT/D(DMA)@DOX for different incubation times. And the cells without any treatment were used as control. After co-incubation for predetermined time, the cells were washed with PBS for three times and the media were replaced with DCFH-DA (10 μM) at 37 °C for 30 min. Finally, all the cells were observed by fluorescence microscopy and the green fluorescence intensity was measured with a microplate reader (ELIASA of Perkin Elmer) at 490 nm.

### Cell apoptosis assay

To investigate the apoptotic effect of different drug formulations, A549 cells were treated with free DOX, free TOS, DOX/TOS, PPT/D(SA), PPT/D(DMA), PPT/D(SA)@DOX and PPT/D(DMA)@DOX. The concentration of DOX and TOS was set as 0.5 and 0.75 μg/mL. The extent of apoptosis in A549 was evaluated by flow cytometry (FCM) (ESP Elite, Beckman-Coulter, Miami, FL) analysis using FITC-conjugated AnnexinV/propidium iodide (PI, BD PharMingen) staining, following the manufacturer’s instructions. Both early apoptotic (Annexin V-positive, PI-negative) and late apoptotic (Annexin V-positive and PI-positive) cells were included in cell death determinations.

### Cell uptake

A549 cells were seeded in the glass bottom dishes at a density of 1.0 × 10^5^ cells per well for 24 h. Then all the cells were incubated with PPT/D(SA)@DOX or PPT/M(DMA)@DOX (both containing 2.5 μg/mL DOX) for 2 and 4 h at pH 7.4 and 6.8, respectively. Thereafter, the media were removed and the cells were washed with PBS to remove the extracellular micelles. The cell nuclei were stained by DAPI according to the standard protocol provided by the supplier. At last, the cellular uptake of samples was visualized under fluorescence microscopy. To quantitatively investigate cellular uptake, A549 cells were treated with with PPT/D(SA)@DOX or PPT/M(DMA)@DOX (both containing 2.5 μg/mL DOX) for 2 and 4 h at pH 7.4 and 6.8, respectively. After detachment of cells, the mean fluorescence intensity of cells was detected by FCM.

### Cell viability study

The cytotoxicity of free DOX, free TOS, PPT/D(SA), PPT/D(DMA), PPT/D(SA)@DOX and PPT/D(DMA)@DOX were evaluated by the MTT assay. A549 cells were seeded in 96-well plates at a density of 5000 cells per well in 100 μL DMEM containing 10% FBS and cultured for 24 h at 37 °C. Then the cells were treated with 100 μL culture medium containing fixed amount of micelles for 48 h. After that the medium was replaced with 200 μL of fresh DMEM and 20 μL MTT (5 mg/mL in PBS) and incubated for another 4 h. Then the medium was removed and 200 μL DMSO was added. The optical absorbance was measured at 450 nm of each well using a microplate reader. To detect the pH-responsive charge conversion, the cytotoxicity of PPT/D(SA)@DOX and PPT/D(DMA)@DOX was analyzed at pH 7.4 and 6.8. The cell viability (%) was determined by comparing the absorbance at 450 nm with control wells containing only cell culture medium. All the cytotoxicity tests were conducted in triplicate.

3 $${\text{Cell viability }}\left( \% \right) = \frac{{{\text{OD}}_{\text{sample}} - {\text{OD}}_{\text{blank}} }}{{{\text{OD}}_{\text{control}} - {\text{OD}}_{\text{blank}} }} \times 100\%$$

### Biodistribution

To investigate the biodistribution of blank micelles, tumor-bearing mice were intravenously injected with saline, Cy5-labelled PPT/D(SA) and PPT/D(DMA). At determined time points (12, 24 and 48 h), mice were sacrificed and the major organs, including tumors, were collected. The fluorescent images of these tissues were taken on an infrared imaging system (Caliper, XenoFluor 750).

### In vivo antitumor study and histochemistry analysis

Nude mice (5–6 weeks old) were purchased from Beijing Institution for Drug Control, China. A549 cell tumor-bearing mice model was established by subcutaneous injection of A549 cells (2 × 10^6^) into the right axilla of each mouse. In vivo/ex vivo imaging and biodistribution experiments were performed at day 10 after tumor cell injection, by which time tumors had grown to 0.8 cm in diameter. At that time, A549 cell tumor-bearing nude mice were intravenously injected with saline, free DOX, PPT/D(SA)@DOX and PPT/D(DMA)@DOX micelles at a dose of 0.5 mg/kg DOX. The mice were sacrificed after 24-h post-injection. At 24-h post-injection, the mice were sacrificed, and the hearts, livers, spleens, lungs, kidneys, and tumors were excised to directly observe the fluorescence distribution. The emission fluorescence was collected from 450 to 700 nm, with the 455 nm excitation filter used.

Moreover, when the tumor volume reached 50 mm^3^, A549 tumor-bearing mice were divided into 4 groups randomly (n = 4) and treated with PBS, free DOX, PPD(DMA)@DOX, TOS/PPD(DMA)@DOX, PPT/D(SA)@DOX and PPT/D(DMA)@DOX (fixed concentration of DOX at 2 mg/kg) through intravenous injection at day 0, 3, 6 and 9 respectively. For TOS/PPD(DMA)@DOX treatment, TOS at a dose of 1 mg/kg [27] was used in combination with PPD(DMA)@DOX.

Tumor volumes and body weights of all the mice were recorded every 2 days. Tumor volumes (V) and body weights were measured to evaluate the antitumor activity and systemic toxicity. Tumor volume (V) was calculated using the following formulas:

4 $${\text{V}} = {\text{ a}} \times {\text{b}}^{ 2} / 2$$

where a and b were major and minor axes of the tumors measured by a caliper, respectively and all the mice were sacrificed at day 16. Tumors were dissected, washed and used for histology analysis. Cell state in tumor tissue was analyzed by hematoxylin–eosin (H&E) staining. Simultaneously, to evaluate the levels of apoptosis in tumor areas, tumor tissues were stained by terminal deoxynucleotidyl transferase-mediated dUTP nick-end labeling (TUNEL) according to the manufacturer’s protocol (Roche, Penzberg, Germany). Ki-67-stained sections were observed under a microscope (X51 Olympus; Olympus Corp. Tokyo, Japan).

### Statistical analysis

The results were presented as mean ± standard deviation (SD). Statistical significance was analyzed using Student’s t-test.

## Results and discussion

### Synthesis and characterization of micelles

**Scheme 2:**
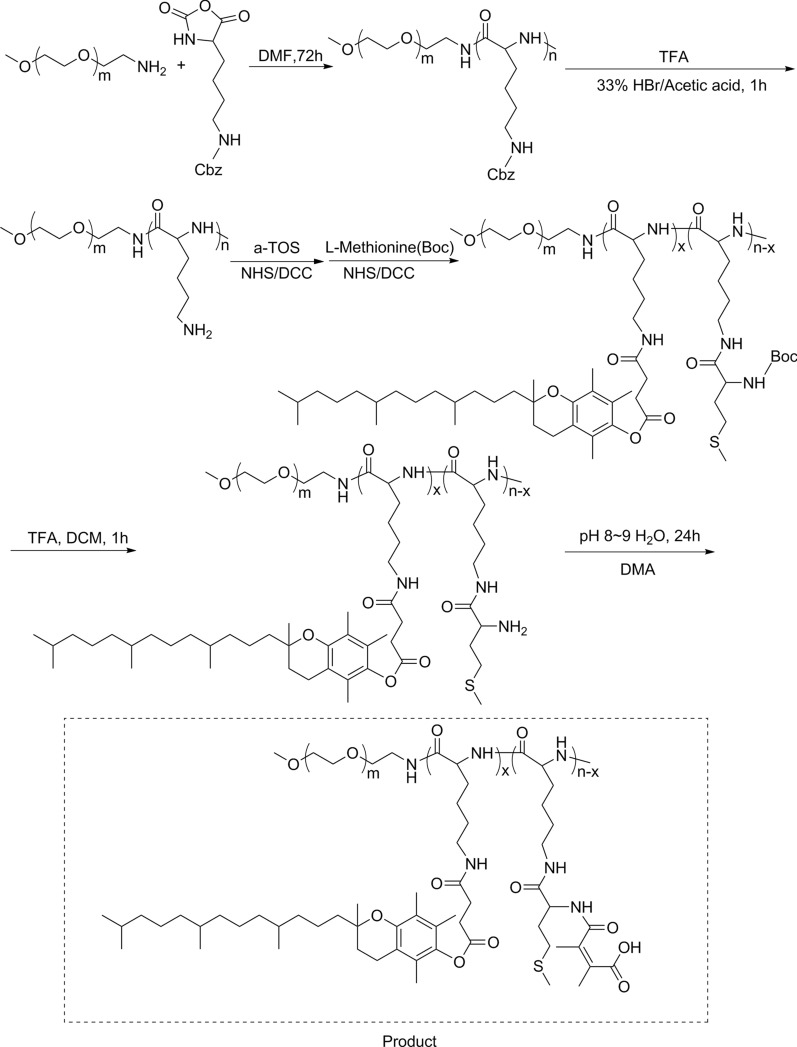
Synthesis routes of PPT/D(DMA)

**Fig. 1:**
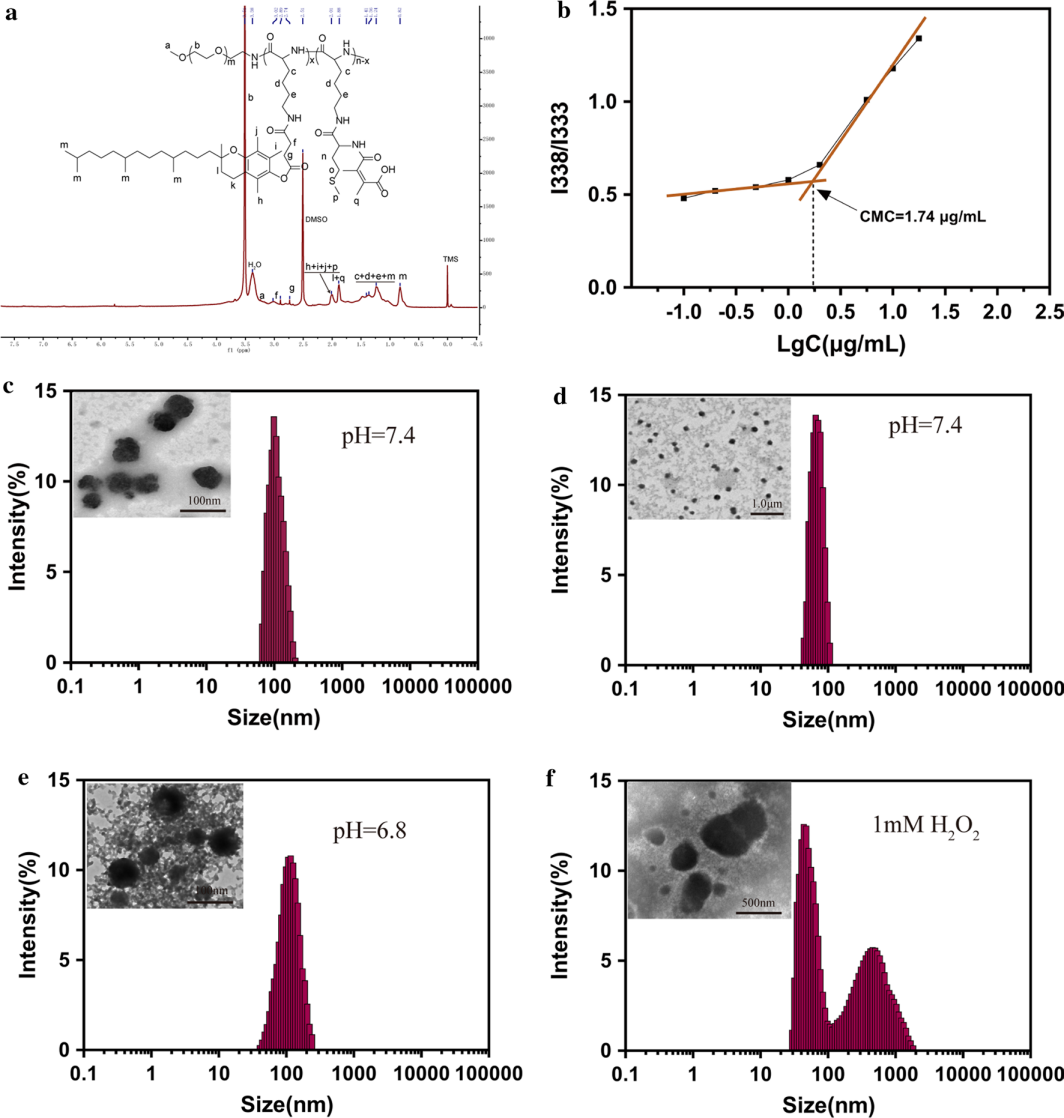
**a** ^1^H-NMR spectra of PPT/D(DMA) in DMSO-*d*_6_. **b** Dependence of excitation fluorescence intensity ratio of pyrene (I338/I333) on the logarithmic concentration of PPT/D(DMA). **c** DLS and TEM images of PPT/D(DMA) blank micelles in PBS (pH 7.4). **d** DLS and TEM images of PPT/D(DMA)@DOX micelles in PBS (pH 7.4). **e** DLS and TEM images of PPT/D(DMA)@DOX micelles at pH 6.8 after 24-h incubation. **f** DLS and TEM images of PPT/D(DMA)@DOX micelles at pH 7.4 with H_2_O_2_ for 6 h

In this study, we synthesized a biocompatible pH/ROS-responsive micelle via ROP polymerization and stepwise chemical grafting reactions as illustrated in Scheme 2. Successful synthesis of PPT/D(DMA) was confirmed using ^1^H-NMR as shown in the Fig. 1a. PPT/D(DMA) (^1^H NMR; 500 MHz, DMSO-d_6_, ppm): 0.82 (CH_3_– from TOS); 1.00–1.70 (–CH_2_CH_2_CH_2_–CH– from mPEG and CH_3_– from TOS); 1.88 broad (–CH_2_– from chromanol ring and CH_3_– from DMA); 1.9–2.1 (CH_3_– from chromanol ring and CH_3_–S– from methionine); 2.74 (g, –CH_2_– from TOS); 2.80-2.90 (f, –CH_2_– from TOS); 3.51 (–CH_2_CH_2_–O– from mPEG). The repeating units in PPT/D(DMA) were 1:30:6:24 for mPEG, PLL, TOS and DMA, respectively, as calculated from the ^1^H-NMR results. *M*_n_ of PPT/D(DMA) calculated by ^1^H-NMR was 22.1 kDa, similar with the data of 23,000 g/mol measured by GPC with polydispersity of 1.25. The empty micelle and DOX-loaded micelles (denoted as PPT/D(DMA) and PPT/D(DMA)@DOX) were prepared with the dialysis method. The PPT/D(DMA) block polymer was dissolved in DMSO, then the solvent was gradually removed for self-assembly through solvent exchange processes via dialysis. Fluorescence study indicated that the resulting PPT/D(DMA) micelle had a relatively low critical micelle concentration (CMC) of 1.74 μg/mL (Fig. 1b). Average size of blank micelle and DOX-loaded micelle was measured as shown in Table 1 and Fig. 1c, d. The mean diameter of empty micelle was found to be 98.1 ± 4.5 nm. When DOX was encapsulated in the nanocarriers in proportion of 10%, the mean size of micelles was decreased to 84.3 ± 3.6 nm with a particle size distribution of PDI = 0.108. The LC and EE were calculated to be 9.64% and 96.4%, respectively.

**Table 1:** Investigation in LC and EE of DOX micelles

Number	DOX/polymer ratio(w/w)	Designed LC %	Measured LC %	EE %	Size (nm) (PDI)^a^
1	0/100	0	–	–	98.1 (0.165)
2	10/90	10	9.64	96.4	84.3 (0.108)

^a^Average diameter and PDI of the nanoparticles measured by DLS

To investigate the sensitivity of micelles to pH and ROS, TEM and DLS were used to study the morphology and size change of PPT/D(DMA)@DOX micelle in response to pH and ROS stimuli. The micelle was characterized in terms of size and surface morphology at pH 6.8 after 24-h incubation and with H_2_O_2_ for 6 h as shown in Fig. 1e, f. At pH 7.4, the average size of the PPT/D(DMA)@DOX was 84.3 ± 3.6 nm, which increased to 116.2 ± 6.3 nm at pH 6.8. This might resulted from the swell of exposed amine from methionine segments with positive charge after the cleavage of DMA groups from the micelle at pH 6.8 [28]. Similarly, the TEM image revealed increase in the size at pH 6.8. Moreover, PPT/D(DMA)@DOX exhibited a disintegrated morphology in the existence of H_2_O_2_. The destruction of the micelle structures was determined with DLS and two peaks containing a large aggregated form were observed, indicating the high sensitivity of the micellar system to ROS. The underlying mechanism was that thioether groups in methionine segments were changed to hydrophilic sulfoxide groups by ROS and then were induced the disassembly of micelle [20, 29].

### pH and ROS-responsive property and drug release

**Fig. 2:**
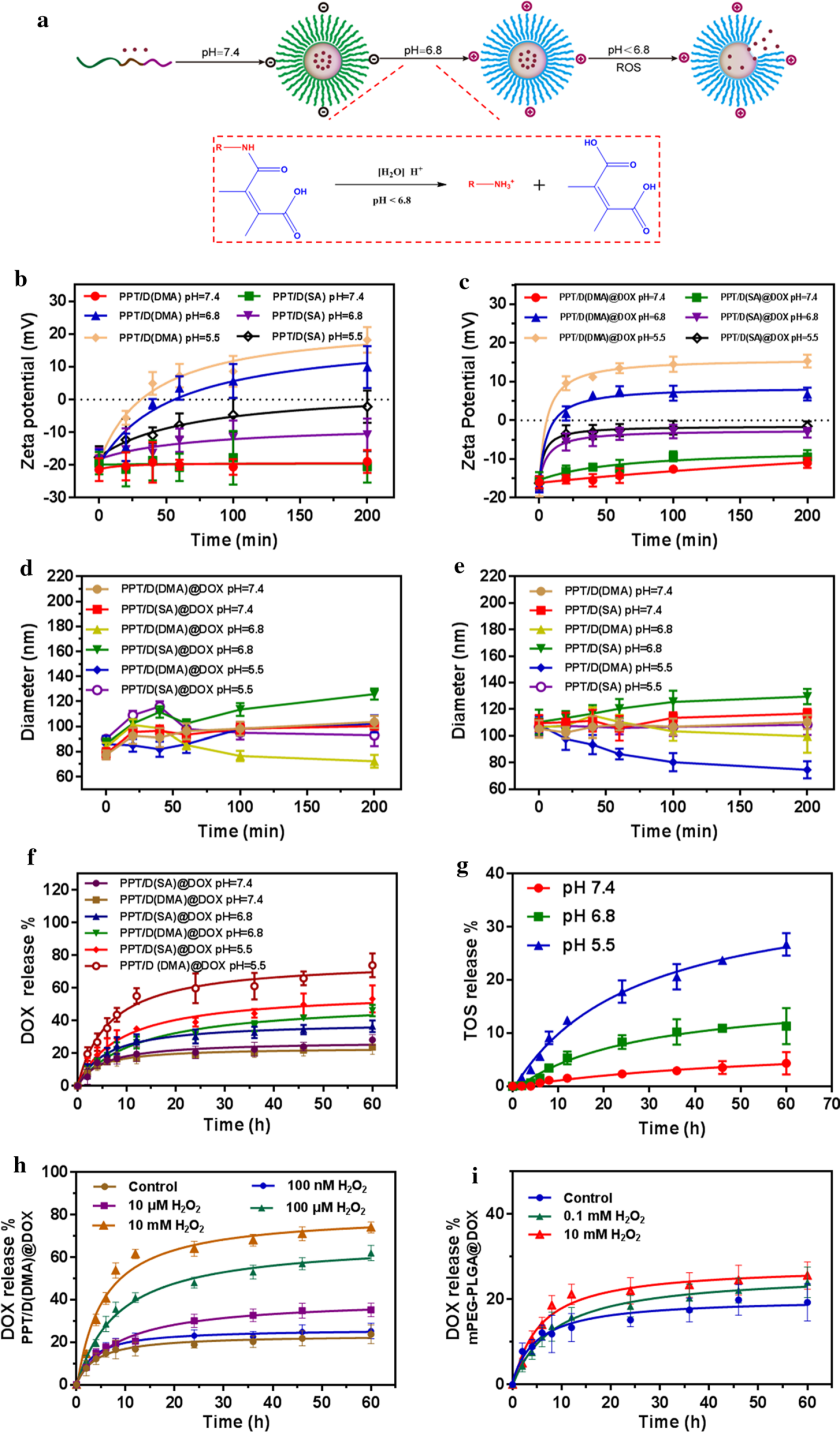
**a** Illustration of the preparation procedure and pH/ROS-dual sensitivity of PPT/D(DMA)@DOX. The micelle took out DMA at pH 6.8 to expose the positive charges, and the release of DOX was triggered quickly by the ROS below pH 6.8. **b** The zeta potential PPT/D(DMA) and PPT/D(SA) after incubation at pH 5.5, 6.8 and 7.4 for 200 min. **c** The zeta potential PPT/D(DMA)@DOX and PPT/D(SA)@DOX after incubation at pH 5.5, 6.8 and 7.4 for 200 min. **d** Diameter of PPT/D(DMA) and PPT/D(SA) after incubation at pH 5.5, 6.8 and 7.4 for 200 min. **e** Diameter of PPT/D(DMA)@DOX and PPT/D(SA)@DOX after incubation at pH pH 5.5, 6.8 and 7.4 for 200 min. **f** Cumulative release of DOX from PPT/D(DMA)@DOX and PPT/D(SA)@DOX micelles at pH 5.5, 6.8 and 7.4. **g** Cumulative release of TOS from PPT/D(DMA)@DOX micelles at pH 5.5, 6.8 and 7.4. **h** Cumulative release of DOX from PPT/D(DMA)@DOX and PPT/D(SA)@DOX micelles at pH 7.4 after treatment with H_2_O_2_ (100 nM,10 μM,100 μM and 10 mM). **i** Cumulative release of DOX from mPEG-PLGA micelles at pH 6.8 after treatment with 0.1 or 10 mM of H_2_O_2_

In this work, the preparation and drug release behavior of PPT/D(DMA)@DOX was used illustrated in Fig. 2a. In order to assess the charge-reversal property and stability of PPT/D(DMA)@DOX in pH 7.4, 6.8 and 5.5, we investigated the changes of zeta potential and size in PBS (pH 7.4, 6.8 and 5.5) in 200 min. When the microenvironment changed from physiological pH (pH 7.4) to slightly acidic conditions (pH 5.5 and 6.8), the negatively charged PPT/D(DMA) turned to positive charge, owing to the conversion reaction between carboxyl groups and amino groups (Fig. 2b). By contrast, the zeta potentials of PPT/D(SA) were negatively charged during the whole incubation. After DOX was loaded in micelles, the changes of zeta potential of PPT/D(DMA)@DOX and PPT/D(SA)@DOX were similar to those of blank micelles as shown in Fig. 2c. Notably, negative charge of PPT/D(DMA)@DOX changed into positive charge after 20 min incubation, which was possibly caused by protonation of DOX. These results suggested that PPT/D(DMA) had a charge-reversal property at pH 6.8, which was attributed to the cleavable amide linkages formed between methionine segments and DMA [30]. To further evaluate the stability in physiological and tumor microenvironment, we measured the size changes of blank micelles and DOX-loaded micelles. As shown in Fig. 2d, the average sizes of PPT/D(DMA) and PPT/D(SA) remained their initial sizes and exhibited a good stability at pH 7.4 within 200 min. With the decrease of pH values, average size of PPT/D(DMA) decreased gradually, while the similar changes of size of PPT/D(SA) were not observed. As shown in Fig. 2e, the average size of PPT/D(DMA)@DOX decreased from 84.3 ± 3.6 nm to 72.2 ± 4.4 nm in 100 min at pH 6.8, whereas that of PPT/D(SA)@DOX was slightly increased in 200 min. Interestingly, PPT/D(DMA)@DOX at pH 5.5 showed larger particle size after 65 min than that at pH 6.8, which was different from PPT/D(SA)@DOX at different pH values. This different phenomenon was probably attributed by the fast detachment of DMA at pH 5.5. The detachment of DMA at pH 5.5 led to the exposure of amines that were protonated immediately, which generated electrostatic repulsion and hence enlarged the core of micelles. The above results demonstrated that PPT/D(DMA)@DOX was negatively charged during the blood circulation (pH 7.4), which reduced the interaction with negatively charged proteins in the blood. When it reached the tumor microenvironment (pH 6.8), the surface of micelle exhibited a charge reversal and turned into positive charge, which would be taken up easily by tumor cells [31]. After endocytosis into tumor cells, low pH values in endo/lysosomes induced the degradation of micelles, which facilitated the drug release.

Subsequently, the quantitative analysis was employed to investigate the pH and ROS-responsive drug release behavior of micelles. As shown in Fig. 2f, the DOX release from PPT/D(SA)@DOX and PPT/D(DMA)@DOX were monitored at different pH values (pH 7.4, 6.8 and 5.5) using the dialysis method. The amounts of DOX released from the micelles were all less than 30% within 60 h at pH 7.4, which validated that these systems were relatively stable and the release rate of DOX was rather slow under physiological conditions. At pH 6.8, the released DOX drugs from PPT/D(SA)@DOX and PPT/D(DMA)@DOX were increased to 36.6% and 46.2% respectively, which were markedly higher than that of pH 7.4. At pH 5.5, the cumulative DOX release from PPT/D(DMA)@DOX and PPT/D(SA)@DOX was 73.9% and 53.4% for 60 h, respectively. These micelles all exhibited significant pH-dependent release behavior. The pH-responsive DOX release from PPT/D(SA)@DOX was mainly attributed to the protonation of DOX at acidic condition, increasing the solubility of DOX. In addition, it worked for PPT/D(DMA)@DOX. However, another reason was responsible for faster drug release from PPT/D(DMA)@DOX. That was because degradation of DMA generated amines that were protonated subsequently, repelling DOX out of the hydrophobic core [32, 33]. TOS release from PPT/D(DMA)@DOX at different pH values was investigated as shown in Fig. 2g. Within 60 h, TOS release was 4.3% at pH 7.4, 11.3% at pH 6.8 and 26.7% at pH 5.5, respectively. Notably, during the beginning 4 h, no TOS can be detected at pH 7.4. Besides, under 0.1 mM and 10 mM H_2_O_2_ conditions, TOS release showed almost the same profiles as that at pH 7.4 (Additional file 1: Figure S7), indicating no ROS-responsive drug release for TOS. As shown in Fig. 2h, around 62.3% DOX released from PPT/D(DMA)@DOX micelle upon treatment with 0.1 mM H_2_O_2_ for 60 h. Furthermore, when the H_2_O_2_ concentration increased to 10 mM, the release percentage reached 74.1%. A small amount of DOX is released when treated with a low concentration of H_2_O_2_. When the H_2_O_2_ concentration fell to 10 μM, the release percentage decreased to 32.4%. While when the concentration of H_2_O_2_ was further reduced to 10 nM, the DOX release rate was only 20.4%, almost no difference from the control group. mPEG-PLGA micelles, made of polymers without methionine, was used as control to verify the effect of methionine on drug release with the presence of H_2_O_2_, as shown in Fig. 2i. The drug release of mPEG-PLGA@DOX was not significantly changed by different concentrations of H_2_O_2_, indicating that methionine groups played a very important role in drug release. These results consistently demonstrated the stability of the system under physiological condition and their pH/ROS responses for controllable DOX release.

### Analysis of the ROS regenerating ability of PPT/D(DMA) in vitro

**Fig. 3:**
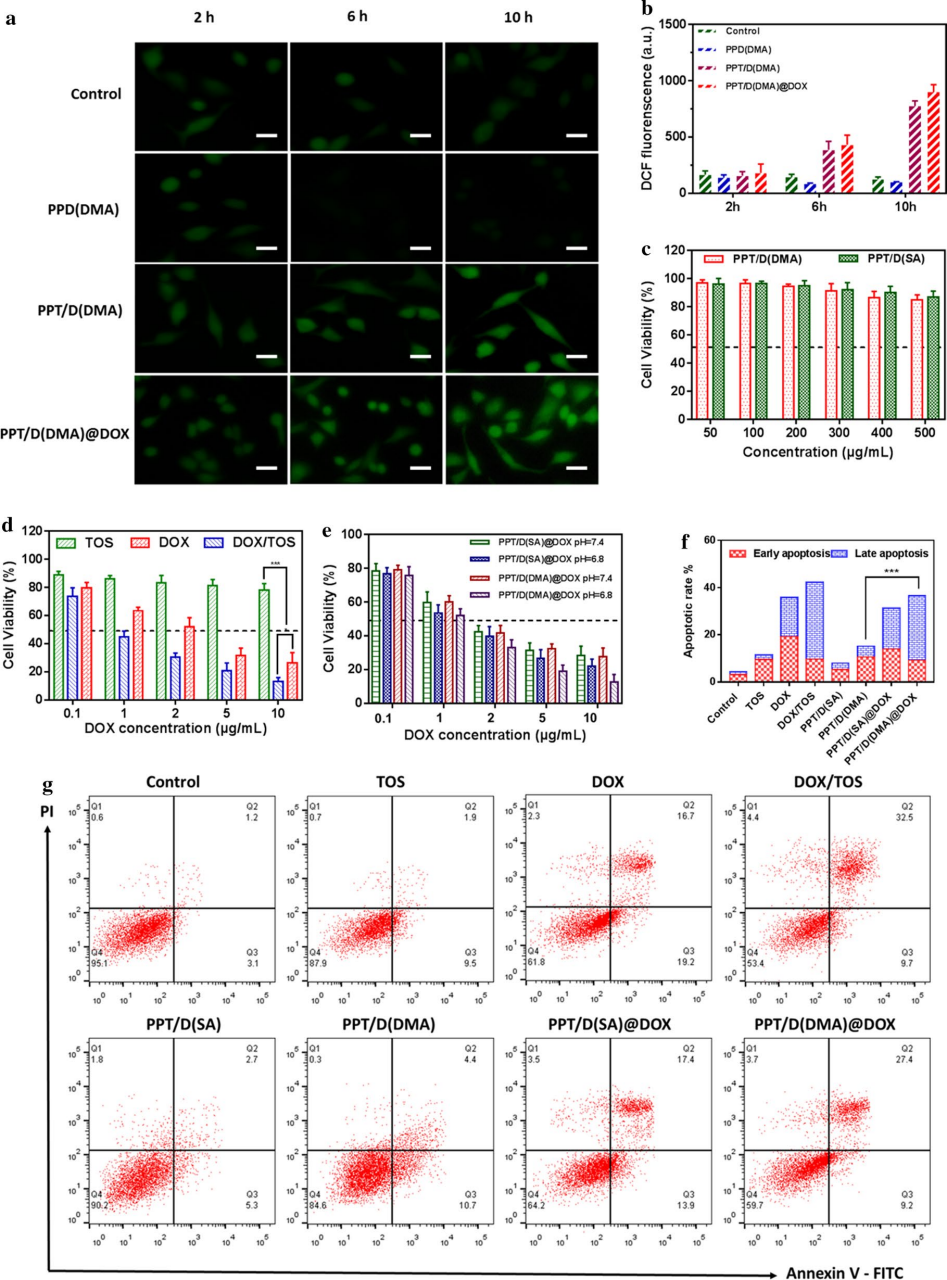
**a** Evaluation of the ROS regenerating ability of PPD(DMA), PPT/D(DMA) and PPT/D(DMA)@DOX in vitro. Fluorescence images of A549 cells treated with different micelles for different time. Scale bar: 20 μm. **b** Quantitative analysis the ROS generation in A549 cells by microplate reader. **c** Cytotoxicity of A549 cells treated with PPT/D(DMA) and PPT/D(SA). **d** Cytotoxicity of A549 cells treated with free TOS, free DOX and TOS/DOX. **e** Cell viability of A549 cells treated with PPT/D(SA)@DOX and PPT/D(DMA)@DOX at various concentrations of DOX at pH 7.4 and 6.8. **f** Apoptosis analysis of A549 cells induced by PBS (control), DOX, TOS, DOX/TOS, PPT/D(SA), PPT/D(DMA), PPT/D(SA)@DOX and PPT/D(DMA)@DOX (equivalent of 2.0 μg/mL DOX and 4.0 μg/mL TOS). **g** Quantification of apoptosis result. All error bars were presented as mean ± SD. *p < 0.05, **p < 0.01

In order to better explore the feasibility of positive feedback strategy to overcome the obstacles in ROS-responsive PPT/D(DMA)@DOX, the prevailing intracellular ROS sensitive probe 20,7-dichlorofluoresceindiacetate (DCFH-DA) was utilized to confirm the ROS generation [34]. Cell permeable nonfluorescent DCFH-DA was rapidly oxidized to dichlorofluorescein (DCF) with green fluorescence by the intracellular ROS. As shown in Fig. 3a, b, the conspicuous green fluorescence of DCF in A549 cells clearly proved the inherent existence of intracellular ROS. When the A549 cells were incubated with PPT/D(DMA) micelles, the green fluorescent signal was noticeably stronger than that without PPD(DMA) micelles, owing to the ROS generating capability of TOS segments, which restrained the bioactivity of mitochondrial respiratory complex II, contributing to the electron transfer to produce ROS from oxygen [13]. Interestingly, PPT/D(DMA)@DOX induced more ROS than PPT/D(DMA). In addition, after prolonging the incubation time, the green fluorescence in A549 cells became stronger. Further, compared to the A549 cells as the control for the same time, after cells were treated with PPT/D(DMA) for 10 h and incubated with DCFH-DA, the fluorescence signal in A549 cells increased approximately eightfolds measured by microplate reader (Fig. 3b). The results above confirmed the intracellular ROS producing ability of TOS segments and also revealed the possibility of PPT/D(DMA) for positive feedback strategy.

### In vitro cytotoxicity

The cytotoxicity of PPT/D(DMA) and PPT/D(SA) was evaluated by MTT assay as shown in Fig. 3c. At all tested concentrations, both micelles showed cell viability over 80%, indicating great biocompatibility. Cell viability of DOX and TOS was shown in Fig. 3d. At the fixed ratio of DOX/TOS, DOX showed higher cytotoxicity than TOS. Interestingly, the cytotoxicity of DOX was further enhanced by TOS.

To investigate the cytotoxicity of PPT/D(SA)@DOX and PPT/D(DMA)@DOX at pH 7.4 and 6.8, the MTT assay in A549 cells was performed as shown in Fig. 3e. All the groups presented a DOX concentration-dependent growth inhibition effect on A549 cells. Cytotoxicity at pH 6.8 treated with both micelles was higher than that at pH 7.4. Notably, both PPT/D(SA)@DOX and PPT/D(DMA)@DOX at pH 7.4 exhibited no significant difference in cell viability. However, at pH 6.8, cytotoxicity of PPT/D(DMA)@DOX was significantly higher than that of PPT/D(SA)@DOX, especially at high concentration of DOX. Moreover, half-maximal inhibitory concentration (IC_50_) of PPT/D(SA)@DOX and PPT/D(DMA)@DOX micelles for 48 h was 1.08 μg/mL and 0.75 μg/mL respectively at pH 6.8 meaning that the pH-sensitive micelles with charge reversal promoted the phagocytosis effect of tumor cells and enhanced the DOX accumulation in tumor cells [35].

### Apoptosis assay

In vitro antitumor activities were analyzed by apoptosis assay using FITC Annexin V and Propidium Iodide (PI). As shown in Fig. 3f, g, in the control groups, negligible apoptotic cells were confirmed in the A549 cells. After 24 h, the apoptosis percentage of free DOX and free TOS was 35.9% and 11.4%, respectively, lower than that of DOX/TOS (42.3%). Blank micelles, PPT/D(DMA) and PPT/D(SA), exhibited the apoptosis rate of 15.1% and 8%, respectively. PPT/D(DMA)@DOX and PPT/D(SA)@DOX exhibited the apoptosis rate of 36.6% and 31.3%, respectively. PPT/D(DMA)@DOX micelles had relative high percentages of apoptotic cells, confirming the enhanced antitumor efficacy of PPT/D(DMA)@DOX system [36].

### Cellular uptake study

**Fig. 4:**
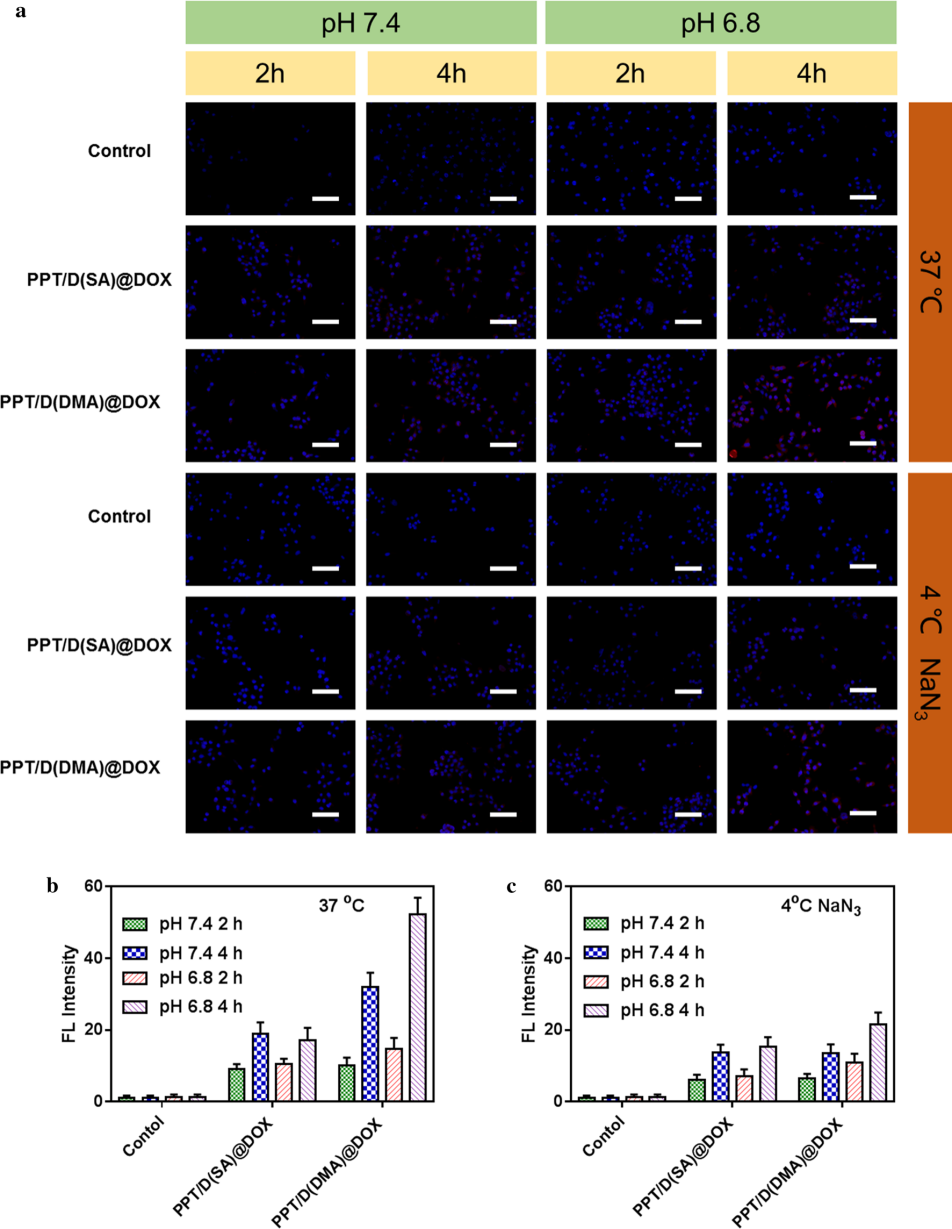
**a** Fluorescence images of A549 cells incubated with PPT/D(SA)@DOX and PPT/D(DMA)@DOX with the same concentration of 2.0 μg/mL in 37 °C and 4 °C for 2 h and 4 h at pH 7.4 and 6.8. Scale bar: 50 μm. Blue: DAPI; red: DOX. Quantitative analysis of flow cytometry results of cellular uptake of PPT/D(SA)@DOX and PPT/D(DMA)@DOX in 37 °C (**b**) and 4 °C (**c**)

To explore the mechanisms of exerted cytotoxicity and apoptosis effect of PPT/D(SA)@DOX and PPT/D(DMA)@DOX, we used the cellular uptake of PPT/D(SA)@DOX and PPT/D(DMA)@DOX on A549 cells at pH 7.4 and 6.8 by fluorescence microscopy. As demonstrated in Fig. 4a, upon cultured with PPT/D(SA)@DOX and PPT/D(DMA)@DOX at pH 6.8, cells exhibited much stronger DOX fluorescence compared to those at pH 7.4, especially after 4-h incubation. In the PPT/D(SA)@DOX group, slight red fluorescence was observed at 2 h after incubation and fluorescence intensity was increased after 4-h incubation. Meanwhile, the more fluorescence in cytosol and nucleus was observed. Moreover, PPT/D(DMA)@DOX group could rapidly accumulate in the cytosol after 2-h incubation, which was revealed by the slight red fluorescence. After 4 h, the more fluorescence nucleus was observed. By contrast, PPT/D(DMA)@DOX showed higher fluorescence intensity than PPT/D(SA)@DOX, indicating that PPT/D(DMA)@DOX had the strongest cellular uptake effect at pH 6.8, which was in accordance with the MTT results. To quantitatively evaluate the cellular uptake, A549 cells were treated with PPT/D(SA)@DOX and PPT/D(DMA)@DOX and analyzed by flow cytometry as shown in Fig. 4b These data demonstrated that the charge-reversal obviously enhanced cellular uptake in the tumor microenvironment [37].

To investigate whether cell uptake of nanomaterials was ATP-dependent, A549 cells were pretreated with 0.1% NaN_3_ culture medium without serum for 1 h as a control. The results showed that low temperature (4 °C) and pre-treatment of cells with 0.1% NaN_3_ culture medium without serum did significantly reduce the fluorescence intensity of intracellular materials, indicating that the nanoparticles did enter the cells through cell uptake. At the same time, the above treatment did not completely inhibit the uptake of nanoparticles into the cells, which was due to the presence of exogenous ATP and sugars in the serum-free medium to provide energy for cell uptake [38].

### Ex vivo imaging

**Fig. 5:**
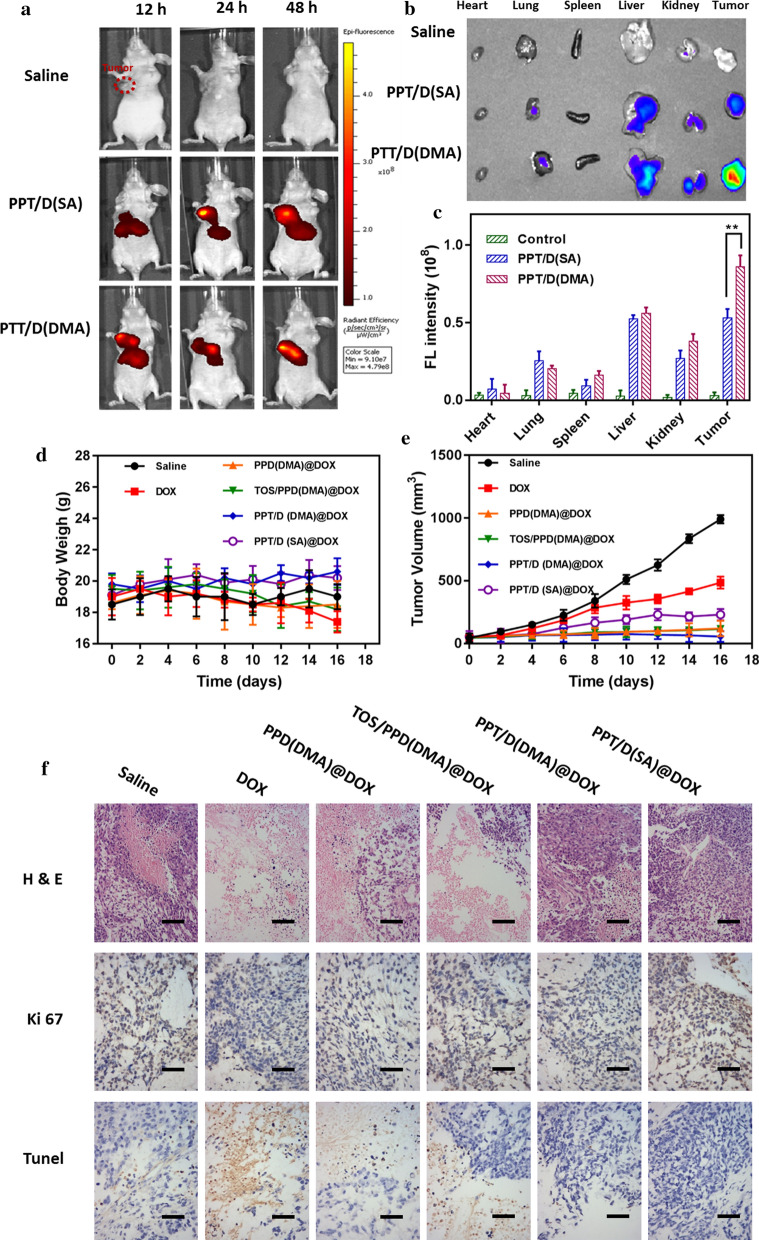
*Ex vivo* representative fluorescence imaging of mice (**a**) and various organs (**b**) from mice treated with saline, Cy5-labelled PPT/D(SA) and PPT/D(DMA), with the quantitative analysis (**c**). Body weight (**d**) and tumor volume (**e**) of mice treated with different groups. **f** Images of H&E, TUNEL and Ki67 of tumor sections, respectively. Scale bar: 100 μm. Error bars were presented as mean ± SD (n = 4). *p < 0.05, **p < 0.01

To verify the biodistribution of PPT/D(SA) and PPT/D(DMA) during systemic circulation, the mice were injected intravenously with Cy5-labelled PPT/D(SA) and PPT/D(DMA). After 12, 24 and 48 h, mice were exposed on a photo imaging system. As shown in Fig. 5a, both PPT/D(SA) and PPT/D(DMA) effectively accumulated in tumor tissues, indicating good tumor-targeting. After mice were sacrificed, various organs and tumors were isolated to image ex vivo. As showed in Fig. 5b, weaker fluorescence in the liver and stronger fluorescence in the tumor was observed in PPT/D(SA) and PPT/D(DMA) micelles group, indicating that both micelles significantly improved the tumor targeting and accumulation, on account of the (enhanced permeability and retention) EPR effect. In addition, the biodistribution of PPT/D(DMA)@DOX and PPT/D(SA)@DOX was also evaluated as shown in Additional file 1: Figure S6. Free DOX was mainly metabolized via liver and kidney, leading to a high accumulation in these two organs. Tumor fluorescence intensity of PPT/D(DMA)@DOX group was distinctly much stronger than those of other groups, which further demonstrated the potency of charge-reversal in improving the accumulation and prolonging the retention of DOX at the tumor site [39].

### In vivo tumor therapy test

A xenograft model was constructed by implanting BALB/c nude mice with 2.0 × 10^6^ A549 cells. Body weights and tumor volumes were measured every 2 days, as shown in Fig. 5c, d. Different treatments induced various extents of tumor growth suppression compared to control (saline). PPD(DMA)@DOX exhibited the similar tumor growth inhibition with TOS/PPD(DMA)@DOX. The non-specific distribution of free TOS was responsible for the inefficiency. The tumor growth inhibition of PPT/D(DMA)@DOX groups was much higher than that of PPT/D(SA)@DOX. These results indicated the synergistic effect of TOS and DOX. The body weight loss was an important indicator for evaluating drug-induced toxicity. Treatment with free DOX resulted in the greatest body weight loss (8.4%) compared with DOX-loaded micelles (~ 5.2%), which revealed that free drug had significant treatment-related toxicities. More importantly, PPT/D(DMA)@DOX induced the greatest tumor suppression among all groups without body weight loss, and the tumor size was significantly reduced after treatment, indicating the superior antitumor effects. The reasons were elucidated as followed. Firstly, the long-circulating PEG layer and negative charged surface of PPT/D(DMA)@DOX system prolonged the circulation time and increased nanocarrier accumulation at tumor sites through EPR effect [40]. Secondly, tumor acidity-activating charge conversion effectively improved cell uptake of PPT/D(DMA)@DOX. Moreover, after internalization, the endogenous ROS would induce micelle disassembly and drug release, and the exposed TOS segments could further produce ROS for amplifying micelle disassembly and drug release [41, 42]. Above reasons contributed to the superior therapeutic efficacy of PPT/D(DMA)@DOX system.

H&E and Ki67 with immunofluorescence staining were performed to further confirm the enhanced antitumor activity of the PPT/D(DMA)@DOX system based on proliferation activity. As shown in Fig. 5e, PPT/D(SA)@DOX and PPT/D(DMA)@DOX groups inhibited proliferation markedly than free DOX, as revealed by the distinct cell shrinkage and chromatin condensation in H&E observation and fewer magenta dots co-located with nuclei in Ki67 images. Meanwhile, the proliferation of mice treated with PPT/D(DMA)@DOX was lowest, which was consistent with TUNEL assay for tumor tissues. PPT/D(DMA)@DOX group displayed the highest level of TUNEL expression among all groups, meaning the severe cell apoptosis. The results demonstrate that PPT/D(DMA)@DOX could effectively deliver DOX to tumor and induce tumor cells apoptosis with high efficiency in vivo.

## Conclusions

In summary, we developed a pH/ROS-responsive micelle drug delivery system with charge reversal and self-amplifiable drug release for tumor therapy. The micelles with negatively charged surface in blood had a great ability of prolonging circulation time; the charge reversal occurred when micelles were exposed in acidic conditions, resulting in excellent cell membrane penetrating. It was found that owing to the ROS-responsive thioether groups, this designed nano-system disassembled and delivered the drug to tumor cells and produced the cell toxicity. Moreover, exposed TOS segments led to the augmented concentration of intracellular ROS and accelerated release of DOX. Due to its unique advantages such as efficient cellular uptake and triggering targeted drug release, this designed system with charge reversal will exhibited great potential for achieving better therapeutic effects in cancer treatment.

## Supplementary information

**Additional file 1: Figure S1.** ^1^H-NMR spectra of PEG-*b*-PLL(Z) and PEG-*b*-PLL. **Figure S2.** ^1^H-NMR spectra of N-(tert-Butoxycarbonyl)-l-methionine (D(Boc)) and methoxyl poly(ethylene glycol)-*block*-Poly(l-lysine)-*graft*- methionine (Boc) (PLM(Boc)). **Figure S3.** ^1^H-NMR spectra of PPT/D(Boc) and PPD(DMA). **Figure S4.** ^1^H-NMR spectra of PPT/D(SA) in DMSO-d6. **Figure S5.** ^1^H-NMR spectra of methionine treated with H_2_O_2_ for 0, 4 and 12 h in D_2_O. **Figure S6.** Ex vivo representative fluorescence imaging (**a**) and quantitative analysis (**b**) of various organs, including tumor tissue, from mice treated with saline, PPT/D(SA)@DOX and PPT/D(DMA)@DOX. **Figure S7.** Cumulative release of TOS from PPT/D(DMA)@DOX micelles at pH 7.4 after treatment with 0.1 and 10 mM of H_2_O_2_.

## Data Availability

All data generated or analyzed during this study are included in the article and additional file. The additional file is available.

## Abbreviations

DMA: Dimethylmaleic anhydride
PPT/D(DMA): Methoxyl poly(ethylene glycol)-*block*-poly(l-lysine)-*graft*-α-tocopheryl succinate and methionine modified with dimethylmaleic anhydride
DOX: Doxorubicin
TOS: α-tocopheryl succinate
PBS: Phosphate buffer saline
TEM: Transmission electron microscopy
ROS: Reactive oxygen species
CMC: Critical micelle concentration
