# Supplementary material for: Dual-responsive doxorubicin-loaded nanomicelles for enhanced cancer therapy
Source: J Nanobiotechnology. 2020 Sep 24;18:136. doi: 10.1186/s12951-020-00691-6 (PMC7517807; doi:10.1186/s12951-020-00691-6)
Supplement: Supplementary file 1 — Additional file 1: Figure S1. 1H-NMR spectra of PEG-b-PLL(Z) and PEG-b-PLL. Figure S2. 1H-NMR spectra of N-(tert-Butoxycarbonyl)-l-methionine (D(Boc)) and methoxyl poly(ethylene glycol)-block-Poly(l-lysine)-graft- methionine (Boc) (PLM(Boc)). Figure S3. 1H-NMR spectra of PPT/D(Boc) and PPD(DMA). Figure S4. 1H-NMR spectra of PPT/D(SA) in DMSO-d6. Figure S5. 1H-NMR spectra of methionine treated with H2O2 for 0, 4 and 12 h in D2O. Figure S6. Ex vivo representative fluorescence imaging (a) and quantitative analysis (b) of various organs, including tumor tissue, from mice treated with saline, PPT/D(SA)@DOX and PPT/D(DMA)@DOX. Figure S7. Cumulative release of TOS from PPT/D(DMA)@DOX micelles at pH 7.4 after treatment with 0.1 and 10 mM of H2O2. [file 12951_2020_691_MOESM1_ESM.docx]

**Additional Information**

**ROS-triggered self-accelerating drug release nanosystem with charge conversion for enhanced cancer therapy**

Xinyi Zhang^1^, Tiantian Zhu^2^, Yaxin Miao^3^, Lu Zhou^3^, Weifang Zhang^1^*.

1.Department of Pharmacy/Respiratory Diseases, The Second Affiliated Hospital of Nanchang University, Nanchang 330006, China.

2.Teaching and Research Office of Clinical Pharmacology, College of Pharmacy, Xinxiang Medical University, Xinxiang, 453003, China.

3.Medical College of Nanchang University，Nanchang, 330031，China.

*Corresponding to Weifang Zhang, e-mail: zhaweifang@163.com, Tel.: +86-0791-86303184.


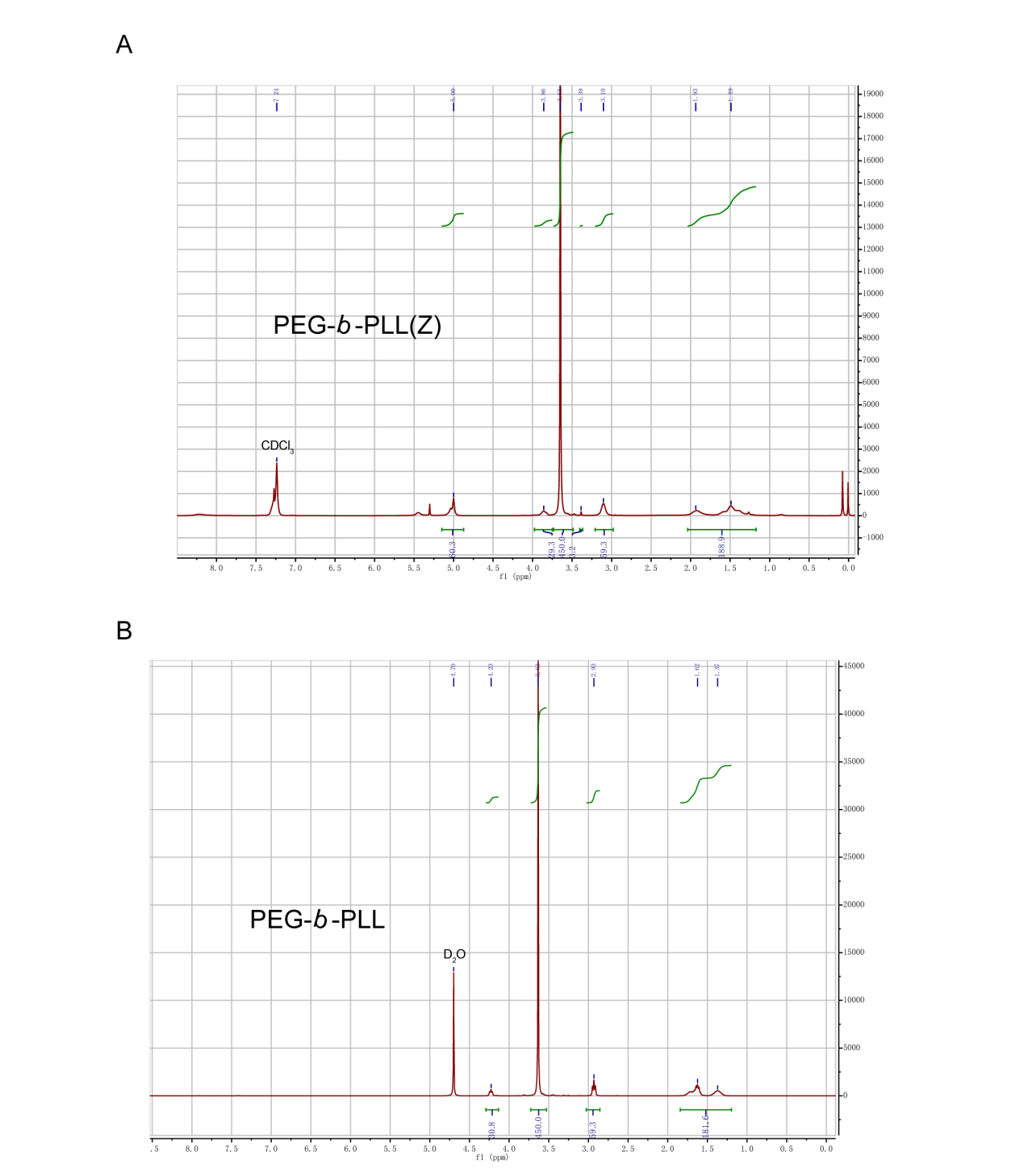


**Figure S1.** ^1^H-NMR spectra of PEG-*b*-PLL(Z) and PEG-*b*-PLL.

PEG-*b*-PLL(Z) (^1^H NMR; 500 MHz, CDCl_3_, ppm): 1.20-1.90 (-CH_2_CH_2_CH_2_-CH-), 3.10 (-CH_2_-NH-), 3.66 (-CH_2_CH_2_-O-), 3.86 (-CH-NH-), 5.00 (-CH_2_-C_6_H_5_), 7.26-7.44 (-C_6_H_5_). PEG-*b*-PLL (^1^H NMR; 500 MHz, D_2_O, ppm): 1.32-1.70 (-CH_2_CH_2_CH_2_-CH-), 2.93 (-CH_2_-NH_2_), 3.63 (-CH_2_CH_2_-O-), 4.23 (-CH-NH-).


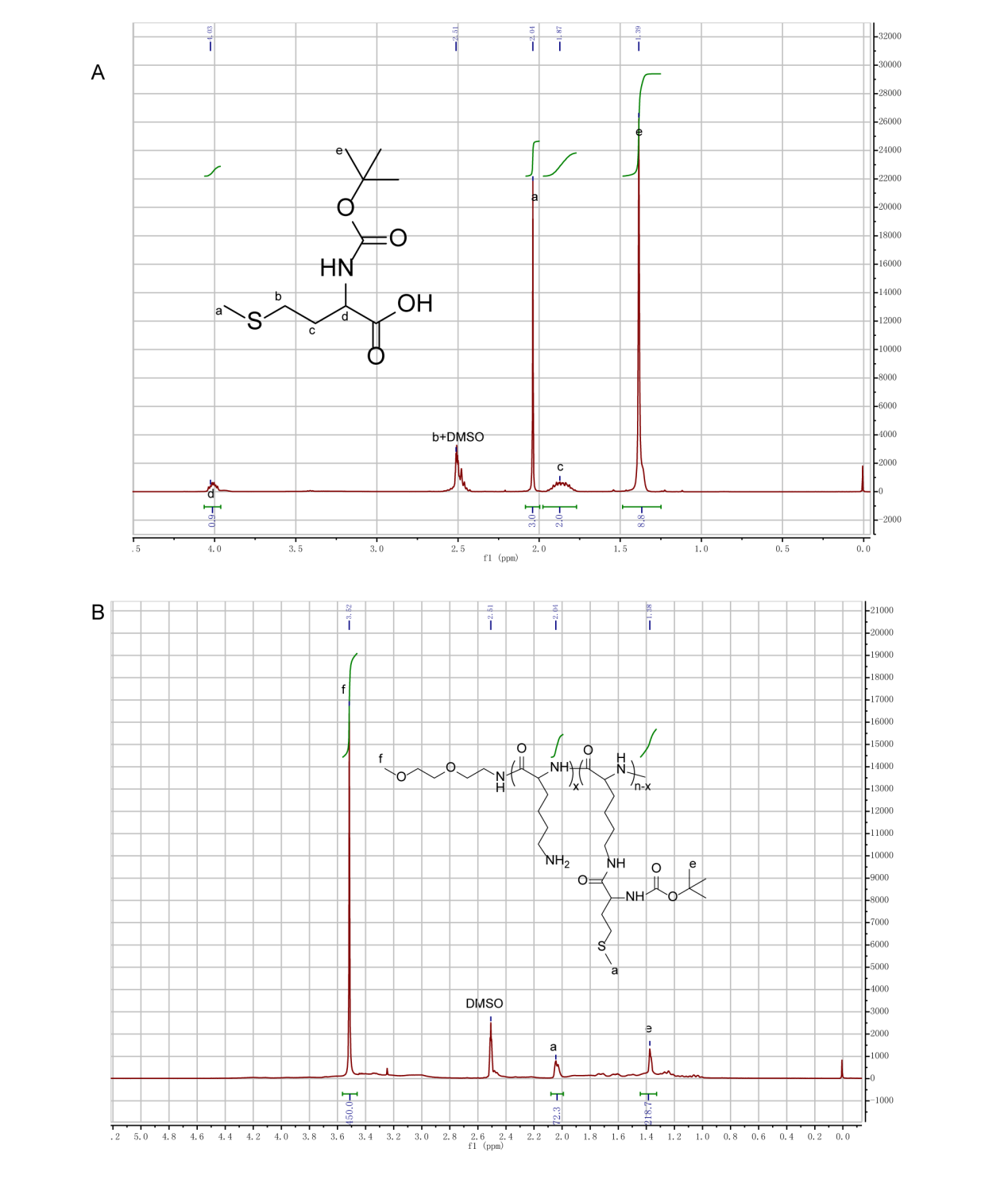


**Figure S2.** ^1^H-NMR spectra of N-(tert-Butoxycarbonyl)-L-methionine (D(Boc)) and methoxyl poly(ethylene glycol)-*block*-Poly(L-lysine)-*graft-* methionine (Boc) (PLM(Boc)).

D(Boc) (^1^H NMR; 500 MHz, DMSO-d_6_, ppm): 1.39 ((CH_3_)_3_C-O-), 1.85-1.90 (-CH_2_-CH-), 2.04 (CH_3_-S-), 2.54 (-CH_2_-S-), 4.03 (-CH-NH-). PLM(Boc) (^1^H NMR; 500 MHz, DMSO-d_6_, ppm): 1.38 ((CH_3_)_3_C-O-), 2.04 (CH_3_-S-), 3.52 (-CH_2_CH_2_-O-).


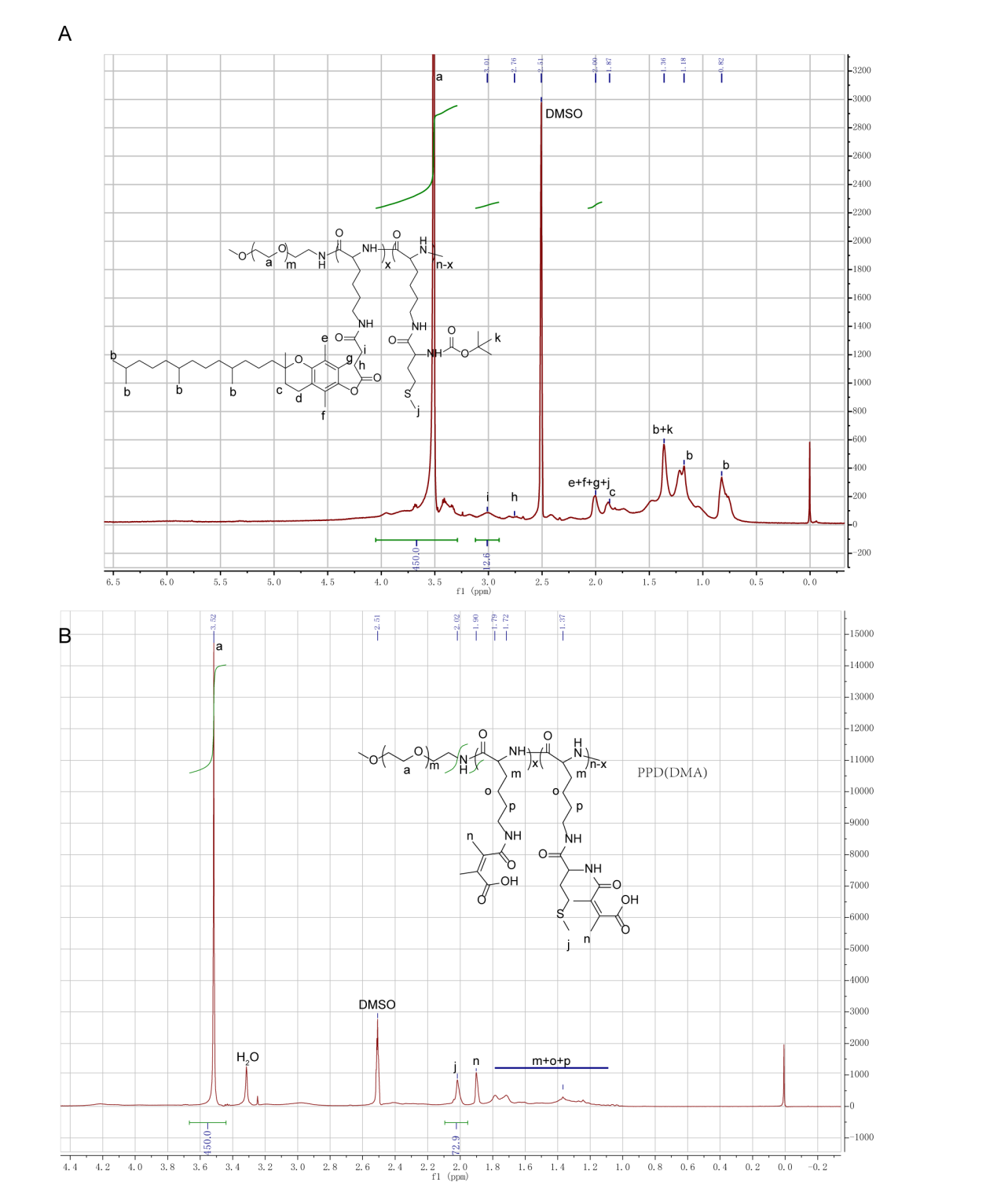


**Figure S3.** ^1^H-NMR spectra of PPT/D(Boc) and PPD(DMA).

PPT/D(Boc) (^1^H NMR; 500 MHz, DMSO-d_6_, ppm): 0.82 (CH_3_- from α-TOS); 1.30-1.40 ((CH_3_)_3_C-O- from methionine and CH_3_- from α-TOS); 1.87 broad (CH_2_- from chromanol ring); 1.9-2.1 (CH_3_- from chromanol ring and CH_3_-S- from methionine); 2.7 (h, -CH_2_- from α-TOS); 3.01 (i, -CH_2_- from α-TOS); 3.4-3.6 (-CH_2_CH_2_-O- from mPEG). PPD(DMA) (^1^H NMR; 500 MHz, DMSO-d_6_, ppm): 1.20-1.90 (-CH_2_CH_2_CH_2_-CH-from mPEG); 1.90 (CH_3_- from DMA); 2.02 (CH_3_-S- from methionine); 3.52 (-CH_2_CH_2_-O- from mPEG).


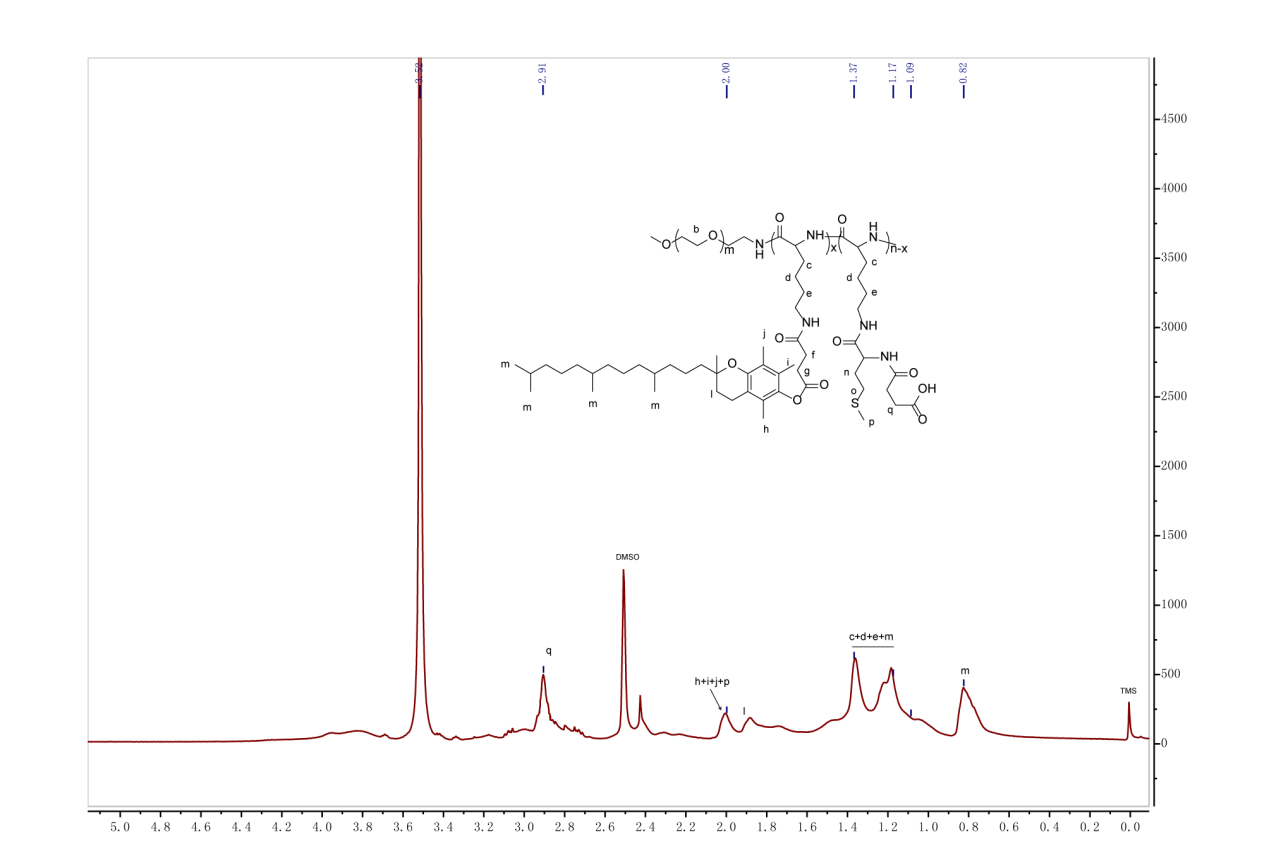


**Figure S4.** ^1^H-NMR spectra of PPT/D(SA) in DMSO-d6.


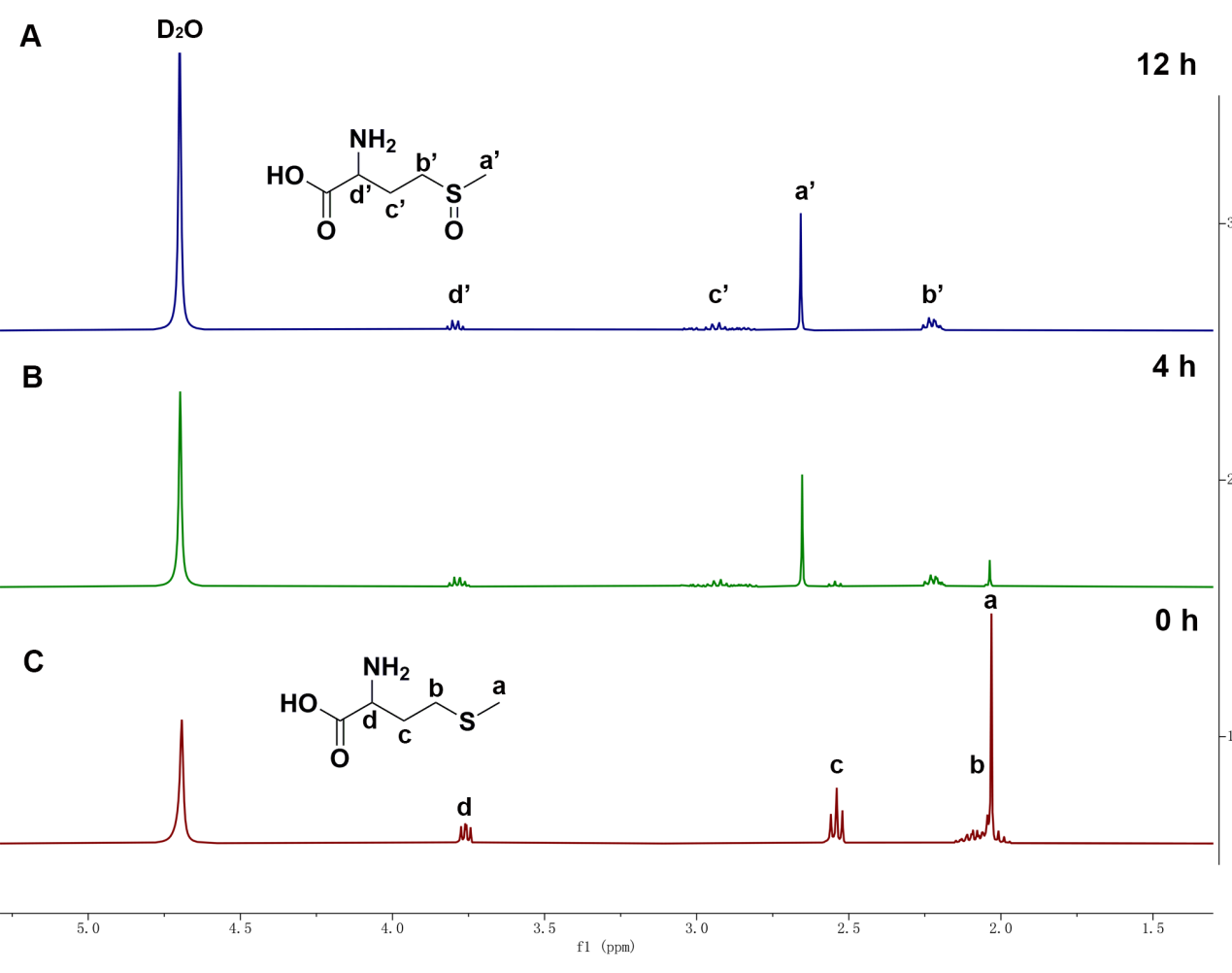


**Figure S5.** ^1^H-NMR spectra of methionine treated with H_2_O_2_ for 0, 4 and 12 h in D_2_O.


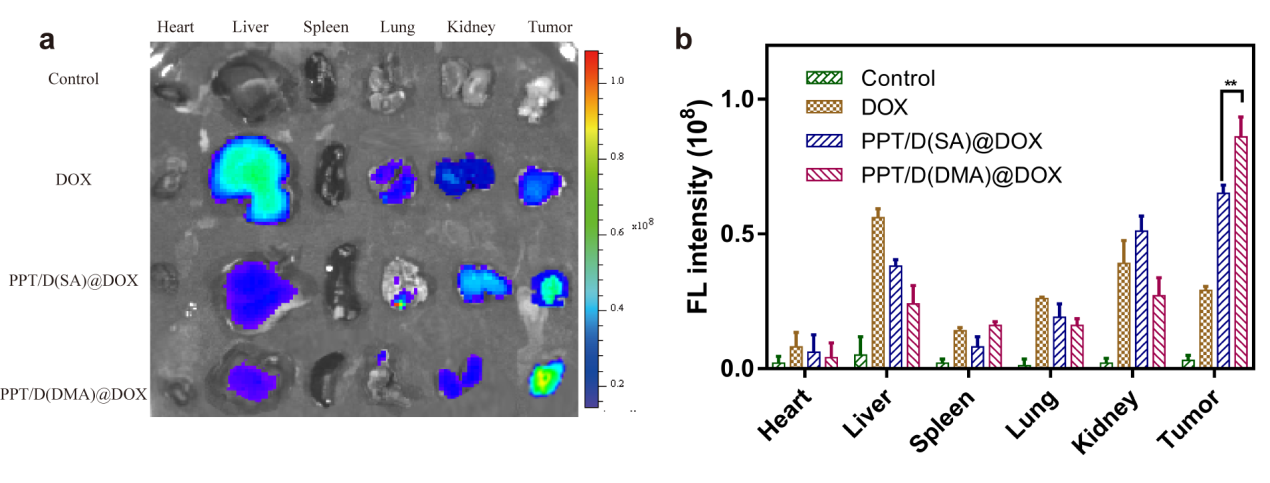


**Figure S6.** *Ex vivo* representative fluorescence imaging (**a**) and quantitative analysis (**b**) of various organs, including tumor tissue, from mice treated with saline, PPT/D(SA)@DOX and PPT/D(DMA)@DOX.

**Figure S7** Cumulative release of TOS from PPT/D(DMA)@DOX micelles at pH 7.4 after treatment with 0.1 and 10 mM of H_2_O_2_.
